# Correlates of alcoholics anonymous affiliation among justice-involved women

**DOI:** 10.1186/s12905-018-0614-0

**Published:** 2018-07-11

**Authors:** Maji Hailemariam, Michael Stein, Bradley Anderson, Yael Chatav Schonbrun, Kelly Moore, Megan Kurth, Fallon Richie, Jennifer E. Johnson

**Affiliations:** 1 Division of Public Health, College of Human Medicine, Michigan State University, Flint, MI USA; 2 Boston University School of Public Health, Health Law, Policy and Management, Boston, MA USA; 3 Butler Hospital, Providence, RI USA; 4 Department of Psychiatry, Yale University, School of Medicine, New Haven, USA

**Keywords:** Alcohol use disorders, Jails, Alcoholics anonymous, Women, Justice-involved

## Abstract

**Background:**

Alcohol use disorder (AUD) constitutes a major public health problem and is associated with a substantial amount of disability and premature death worldwide. Several treatment and self-help options including Alcoholics Anonymous (AA) meetings are available. Nevertheless, factors associated with AA affiliation in some disadvantaged groups such as justice-involved women are not well understood. The purpose of this study is to report on previously unexamined correlates of past year AA affiliation among women in pretrial jail detention.

**Methods:**

The current study used cross-sectional data from 168 women with DSM-5 diagnosis of  AUD in pretrial jail detention. The study examined factors related to women’s concept of self and others (i.e., disbelief that others are trustworthy, lack of autonomy to choose who they interact with, experience of violent victimization, low investment in self-care, higher stress levels, and homelessness) as correlates of past-year AA affiliation, controlling for severity of AUD and demographic factors.

**Results:**

Women who believe that others are inherently trustworthy, women who met less AUD criteria, and women who are older reported more past-year AA affiliation in both univariate and multivariate analyses.

**Conclusion:**

Introducing AA outreach and alternative interventions for younger, less severely addicted women might improve AUD outcomes. Moreover, designing more individualized treatment plan for women who believe others are not trust worthy might help AUD treatment engagement in this population.

**Trial registration:**

NCT01970293, 10/28/2013.

**Electronic supplementary material:**

The online version of this article (10.1186/s12905-018-0614-0) contains supplementary material, which is available to authorized users.

## Background

Alcohol use disorder (AUD) constitutes a major public health problem and is associated with disability and premature death worldwide [1]. According to the WHO, 5.1% of the global burden of disease and injury is attributable to alcohol use problems [2]. Heavy alcohol use is associated with chronic health conditions [3, 4], comorbid psychiatric problems, risky sexual behavior, and high utilization of health care services [5, 6]. According to the 2015 National Survey on Drug Use and Health (NSDUH), approximately 15.1 million adults over the age of 18 and above had AUD [7]; one third (5.7 million) of these were women [2]. Alcohol use is especially prevalent among incarcerated popuations where over 80% of those in jail report alcohol use problems [8].

Since the recognition of AUD as a disorder 60 years ago [9], self-help options have become widespread, particularly 12-step programs [10–12]. Alcoholics Anonymous (AA) is among the most affordable and accessible form of help for justice-involved women [13]. AA could serve as an ideal support for justice-involved women as history of criminal involvement may limit access to treatment and other resources. Incarceration also weakens vital social support which is instrumental in accessing healthcare and other services at reentry [14, 15].

AA attendance has been reported to be efficacious in both community and facility-based samples contributing to higher rates of abstinence and fewer days of drinking [16–18]. Among women leaving jail or prison, attendance of AA meetings after release is associated with a significant decrease in drinking [5]. Despite AA meetings being easier to access than many formal AUD treatment services [19], non-involvement or limited involvement (disaffiliation) with AA meetings is common among justice-involved women [5]. Although AA affiliation incorporates a range of activities such as practicing the steps, leading meetings, having a sponsor or having served as a sponsor and identification with AA groups, attendance of meetings is often considered as the primary marker of affiliation [20]. Members of AA groups often receive soberity chips as markers of the different stages of soberity or as reminders of their commitment to sobriety [21, 22].

Previous studies have examined predictors of 12-step group attendance among persons with AUD. Greater severity of drinking, having previous experience with 12-step groups, and having recovery-oriented social networks have been found to predict greater 12-step group attendance [5, 23–25]. Conversely, younger age [26] lower educational level, lower income, and unemployment generally predict disaffiliation with AA [27]. Some studies suggest that women might react differently to AA than do men due to personal and social difficulties entering into 12-step groups [28]; gender-based social roles and drinking contexts [29] and the belief that AA is geared to men’s needs [30]. Despite their frequent contact with a healthcare provider, women underutilize AUD treatments including AA [31]. Moreover, women might also benefit less because co-occuring conditions such as depression remain unaddressed [16].

Other unexamined factors relating to women’s concept of self and perceived treatment by others may have salience for AA affiliation among justice-involved women. People with AUD, especially women, often experience or anticipate the stigma of alcohol use [32, 33]. Incarcerated women may already be subject to more stigma than are incarcerated men [34]. Anticipated and experienced stigma may result in self-isolation [35] and therefore lead to non-involvement in AUD self-help. Self-stigma, refering to the internalization of negative stereotypes towards individuals with AUD [32], may limit social support and access to resources embedded in social networks [33, 36], potentially including AUD self-help in non justice-involved population.

Interpersonal trust theory deals with behaviors of individuals responding to an immediate situation involving two or more individuals [37]; hence, interactions of women within or outside AA groups fits into these situations. The theory states that an individual’s choice of behavior or decision in a particular situation is affected by their perceived outcomes of the situation or perceived treatment by others [37]. Therefore, an extension of interpersonal trust theory might explain how different interpersonal factors (described below) might influence the behavior of women with AUD leaving jails.

A woman’s ability and willingness to access help in social settings like AA can be affected by interpersonal factors. These include disbelief that others (i.e., people in general) are trustworthy [38], having to interact with people who are risky to their soberiety due to lack of basic needs (eg. needing to trade sex for food or shelter, or staying with an abusive or drug-using partner for lack of options), experience of violent victimization [39], low investment in self-care, and higher stress levels. Women experiencing higher levels of emotional distress attend AUD treatments intermittently, hence, have poorer treatment outcomes [40]. Homelessness may be of particular importance for women seeking treatment for AUD because it often encompasses adverse life-events leading to a decline in the overal quality of life. Having a house also provides stability which is key to accessing social capital and building social networks [41].

Although these factors (i.e., self-stigma, beliefs about others’ trustworthiness, lack of autonomy, violent victimization, self-care, stress, and homelessness), commonly experienced among justice-involved women [42, 43], are plausibly related to women’s ability to participate in AA, none have previously been tested as a correlate of AA affiliation.

The purpose of this study was to examine correlates of past-year AA affiliation among women with heavy alcohol use who are in jail. This study evaluated whether self-stigma, having to interact with people who are risky to their soberiety due to lack of basic needs, low self-care, experience of violent victimization, homelessness, higher stress levels and disbelief that others are trustworthy are associated with low AA affiliation, after accounting for typical correlates of AA attendance (i.e. age, educational level, income, employment status and severity of drinking) [19]. Understanding these correlates could contribute to designing gender-sensitive AUD interventions for justice-involved women who historically have been underrepresented [44] and benefit less from these services.

## Methods

### Participants

The current study used cross-sectional baseline data from an ongoing intervention trial called Community Links to Establish Alcohol Recovery (CLEAR) for women with AUD in pretrial jail detention. The study is being conducted in a jail in Northeast, USA. Details of the methods of the trial is published elsewhere [45]. The recruitment started in late 2013. Participants met DSM-5 criteria for AUD in the 6 months prior to jail detention, were unsentenced, at least 18 years of age, lived within 20 miles of the research office, and did not plan to attend residential substance use treatment after release from jail (so that they would have days in an uncontrolled environment during the post-release follow-up period). Women were ineligible if they were incarcerated for more than 60 days at baseline, plan to attend residential services upon release or if they are sentenced to prison. The study was approved by the Butler Hospital IRB (FWA #00000963) and registered at clinicaltrials.gov (NCT01970293).

### Data collection

The data for this study were collected during pretrial jail detention from all women enrolled in the trial. Affiliation with AA in the past year was the dependent variable.

#### Instruments

##### Sociodemographic factors

A self-report of age, educational level, past year income and employment status were used to assess important sociodemographic indicators.

##### Presence and severity of alcohol use disorder (AUD)

The Structured Clinical Interview for DSM-5 Diagnoses (SCID-5) [46] was used to assess presence and severity of AUD. In the DSM-5, number of AUD criteria is a valid measure of AUD severity. Participants had to meet DSM-5 AUD criteria to be included in the study.

##### Past-year AA affiliation

The AA Affiliation Scale was used to measure affiliation with AA. This scale assesses a range of AA-related behaviors, including meeting attendance, having a sponsor, reading AA literature, and self-identification as an AA member. It has nine items with dichotomous response categories. Most items refer to lifetime AA experiences, such as “Have you had an AA sponsor?” The scale has demonstrated internal consistency (Cronbach’s alpha) of .85 and .84 in treated and untreated sample respectively [47].

*Self-stigma* of AUD was measured by using a self-stigma scale originally developed to measure self-stigma of depression, reworded to refer to alcohol use [48]. Constituent items of the scale are rated by using Likert scale ranging from 1 “completely disagree” to 7 “completely agree”. Items in the scale measure experienced and anticipated stigma in three domains; perceptions towards oneself, other people with AUD and perceived treatment by others. Some of the questions include “People tend to like less those who are receiving professional help for AUD”; “I do not want to live next door to another person who uses alcohol”; “Others view me as morally weak because I use alcohol”; “People’s attitudes about alcohol make me feel worse about myself”. This scale had an internal consistency reliability (Cronbach’s alpha) of .88 in the current sample.

*Limited choice* over one’s network members was assessed using a 7-item scale assessing the degree to which women interact with others they know are risky for their sobriety in order to obtain their basic needs such as housing, transportation, and money. This scale (see Additional file 1) was developed based on a previous qualitative work [49, 50] with incarcerated women undertaken by one of the coauthors (JJ). It had an internal consistency reliability (Cronbach’s alpha) of .88 in this sample.

To measure *self-care*, a nine item assessment tool measuring self-worth and self-care (see Additional file 2) was developed based on one of the coauthor’s (JJ) previous qualitative research with incarcerated women leaving prison [13, 49]. Items included “I take good care of myself,” “I need to take care of people around me before I take care of myself,” “I am worth taking care of,” “I am worth protecting,” “I deserved to be able to take care of myself,” “I deserve to protect myself,” “I neglect myself,” “I put myself in dangerous situations,” and “I take care of my needs,” and were answered on a 5-point Likert Scale from “Strongly agree” to “Strongly disagree.” The scale had an internal consistency reliability (Cronbach’s alpha) of .77 in this sample.

*The Violent victimization* score was a total of positive responses to 10 items describing victimization experiences during a disagreement in the past 3 months. The question stem stated, “In the past 3 months, have you had a disagreement in which someone:” and then specific items included: “Insulted or swore at you,” “Threatened to hit or throw something at you,” “Threw something at you,” “Pushed, grabbed, or shoved you,” “Slapped/smacked you,” “Kicked, bit, or hit you,” “Hit or tried to hit you with something (an object),” “Beat you up,” “Threatened you with a knife or gun,” and “Used a knife or gun on you.” This scale had an internal consistency reliability (Cronbach’s alpha) of .89 in this sample.

*The Housing Status Assessment Guide* [51], was used to assess experiences of homelessness. Response to the question “Do you have a house to go to on the first night after release?” was used to determine housing stability.

*The Perceived Stress* Scale (PSS) was used to measure the degree of stressful life events. The scale was modified to include sources of stress in the areas of employment, financial stress, concerns about safety, pregnancy, loss of family members and separation. It is a 4 items rating scale with scores ranging from 0 to 16. The scale has a good internal consistency with Cronbach’s alpha of .658.

The 10-item *General Trust Scale* (GTS) was used to measure participants’ perceptions of others’ general trustworthiness. Items included, “Most people are basically honest,” “You can’t trust strangers anymore, at work, most people pursue only their own interests,” and “In general, most people behave responsibly toward others.”

### Data analysis

We used the AA Affiliation Scale score as the dependent variable. The primary exposure variables included self-stigma, lack of autonomy to choose who to interact with, low self-care, experience of intimate partner violence, homelessness, higher stress levels, and disbelief that others are trustworthy. We present descriptive statistics to summarize the background characteristics of participants including variables found to be associated with lower AA affiliation and/or attendance in previous studies including age, educational level, income, employment status and severity of drinking. The outcome (AA affiliation) is a nonnegative skewed dependent variable with persons who have never attended an AA meeting at the lower limit of 0. Following the works of others [52–54], we used a generalized linear model with log link, Poisson family error distribution, and robust standard errors. The use of this method with non-count, nonnegative skewed dependent variables has previously been demosntrated [52]. We present unadjusted analyses testing the associations between each variable independently and AA Affiliation. Adjusted analyses enter all variables into a single model predicting AA Affiliation. We also report the 95% confidence interval estimate and *p*-values.

## Results

The study included 168 women in pretrial jail detention. Twelve women refused. Participants averaged 36.0 (± 10.1) years of age, 10.7% were Hispanic, 68.5% were White, 13.7% were Black, and 17.8% identified other racial origins (Table 1). Categories were collapsed to compare non-Hispanic Whites to all racial or ethnic minorities in subsequent analyses. Mean educational attainment was 11.7 (± 2.41) and only 17.3% were employed either part- or full-time. The mean on the eight category measure of legal income was 1.55 (± 1.10, Median = 1); 115 (68.1%) reported legal incomes of less than $10,000 and only 6 (3.6%) reported legal incomes of $30,000 or higher. Twenty women (11.9%) said they did not have a place to stay when released. On average, participants met 7.48 (± 2.39) alcohol use disorder criteria. The mean scores on the AA affiliation scale was 2.32 (± 1.75); 42 (25.0%) women had never attended an AA meeting and were at the lower limit of 0 on the AA affiliation index. Seventy-six (45.2%) had attended at least 1 AA meeting in the past year. Descriptive statistics for other measures and indexes evaluated are also presented in Table 1.

**Table 1:** Background characteristics of the study participants (*n* = 168)

	n (%)	Mean (± SD)	Median	Range
Age		36.0 (± 10.1)	34.5	17–68
Hispanic Ethnicity	18 (10.7%)			
Race				
White	115 (68.5%)			
Black	23 (13.7%)			
Other	30 (17.8%)			
Educational Attainment		11.7 (± 2.41)	12	0–21
Employed Part- or Full-Time	29 (17.3%)			
Income^a^		1.55 (± 1.10)	1	1–8
No Housing After Release	20 (11.9%)			
Number of AUD Criteria Met		7.48 (± 2.39)	8	3–11
AA Affiliation		2.32 (± 1.75)	1.75	0–8
Alcohol Self-Stigma		57.8 (± 18.9)	58	17–112
Ever Attended AA (Yes)	126 (75.0%)			
Attended AA Past Year (Yes)	76 (45.2%)			
Limited choice		6.77 (± 7.96)	3	0–21
Self-Care		31.2 (± 5.48)	31	13–44
Perceived Stress		9.71 (± 3.15)	10	0–16
General Trust		22.5 (± 5.56)	23	10–36
# of Victimizations		3.76 (± 3.04)	3	0–10

^a^Income was assessed as an ordered categorical variable coded 1 = < $10,000, 2 = $10,000 - $19,999, 3 = ($20,000 - $29,999, 4 = ($30,000 - $39,999, 5 = $40,000 - $49,999, 6 = $50,000 - $59,999, 7 = $60,000 - $69,999, and 8 = $70,000 or more

Table 2 provides estimates of unadjusted and adjusted associations of AA affiliation with demographic characteristics and correlates. Since the pattern of statistically significant unadjusted and adjusted associations was consistent, we describe only on the later here. Adjusting for other covariates included in the general linear model, AA affiliation was associated positively and significantly with age (b = 0.036, 95%CI 0.023; 0.050, *p* < .001), number of AUD criteria met (b = 0.071, 95%CI 0.006; 0.135, *p* = .033), and with general trust (b = 0.027, 95%CI 0.003; 0.051, *p* < .030). AA affiliation was not associated significantly with any of the other correlates evaluated in Table 2. Correlations between the variables are presented in Table 3.

**Table 2:** Generalized linear models with log link and poisson family error distribution estimating the unadjusted and adjusted associations of selected covariates with AA affiliation (*n* = 168)

	Unadjusted		Adjusted^b^	
	b (95%CI)^a^	(*p* =)^a^	b (95%CI)^a^	(*p* =)^a^
Age	0.032 (0.020; 0.043)	(< 0.001)	0.036 (0.023; 0.050)	(< 0.001)
NH White	0.174 (−0.159; 0.507)	(0.305)	0.296 (−0.026; 0.618)	(0.072)
Education	0.024 (−0.059; 0.108)	(0.566)	−0.019 (− 0.109; 0.071)	(0.680)
Employed (Part or Full)	−0.35 (− 0.862; 0.161)	(0.179)	− 0.262 (− 0.708 0.185)	(0.251)
Income	− 0.051 (− 0.199; 0.098)	(0.502)	−0.062 (− 0.201; 0.076)	(0.375)
# AUD Criteria Met	0.080 (0.017; 0.143)	(0.013)	0.071 (0.006; 0.135)	(0.033)
Alc. Stigma	0.006 (−0.003; 0.014)	(0.181)	−0.002 (− 0.011; 0.007)	(0.668)
Limited choice	0.002 (−0.017; 0.020)	(0.842)	0.010 (−0.008; 0.027)	(0.288)
Self-Care	−0.009 (− 0.034; 0.017)	(0.509)	−0.003 (− 0.031; 0.024)	(0.818)
Perceived Stress	0.012 (−0.038; 0.061)	(0.643)	−0.000 (− 0.050; 0.049)	(0.993)
Gen. Trust	0.034 (0.007; 0.62)	(0.012)	0.027 (0.003; 0.051)	(0.030)
Housing 1st Night	−0.247 (− 0.558; − 0.063)	(0.118)	−0.273 (− 0.642; 0.094)	(0.145)
# Victimizations	−0.020 (− 0.072; 0.032)	(0.966)	−0.005 (− 0.054; 0.045)	(0.257)

^a^Confidence interval estimates and tests of significance were based on robust variance estimators

^b^The unadjusted models are univariate models predicting AA attendance from each variable separately. The adjusted model includes all variables together

**Table 3:** Product-moment correlations among variables included in multivariate model

	1.	2.	3.	4.	5.	6.	7.	8.	9.	10.	11	12.	13.	14.
1.	1.00													
2.	.34**	1.00												
3.	.08	−.11	1.00											
4.	.06	.18*	.11	1.00										
5.	−.12	−.03	.04	.16*	1.00									
6.	−.05	.06	.14	.24**	.26**	1.00								
7.	.19**	.06	−.03	.02	−.05	.02	1.00							
8.	.10	.22**	−.00	.24**	.02	.15	.30**	1.00						
9.	.02	−.13	.04	.01	.07	.08	.12	.23**	1.00					
10.	−.05	−.04	−.12	−.11	.02	−.01	−.20**	−.26**	−.07	1.00				
11.	.04	.01	.05	.14	.03	.10	.18*	.27**	.16*	−.46**	1.00			
12.	.19**	.07	.19*	.10	.05	.02	−.04	−.04	−.16*	.00	−.05	1.00		
13.	−.09	−.04	.03	−.13	.12	.03	−.03	−.28**	−.07	.11	−.14	.03	1.00	
14.	−.04	−.05	−.06	−.01	−.01	.01	.16*	.14	.22**	−.06	.03	−.10	−.09	1.00

* *p* < .05, ** *p* < .01

*Variables:*

1. AA-Affiliation

2. Years Age

3. Non-Hispanic White

4. Years Education

5. Employed Part- or Full-Time

6. Income

7. Number of AUD Criteria Met

8. Alcohol Self-Stigma

9. Limited Choice

10. Self-Care

11. Perceived Stress

12. General Trust

13. Housing 1st Night When Released

14. Number of Violent Victimizations

## Discussion

AA affiliation is an important predictor of drinking outcomes in people with alcohol use problems. Frequent AA attendance is associated with increased abstinence and fewer days of drinking [18, 55]. This study examined correlates of past-year AA affiliation among women with AUD in pretrial jail detention. Contrary to expectation, some of our correlates (low self-care, higher stigma of AUD, higher perceived stress, homelessness) and predictors from previous studies [23–25, 27], (income, educational attainment, employment status and race), were not significantly associated with AA affiliation in this population. We anticipate that this was because of the uniqueness of the study population (justice-involved). Besides, previous studies reporting these correlates were are also based on population-level or facility-based sample; a different demographic group with its own distrinct characteristics [23–25, 27]. On the other hand, the belief that others are trustworthy, older age and higher AUD severity were significantly associated with higher AA affiliation.

Among those variable newly tested in this study, the belief in the intrinsic trustworthiness of others was significantly associated with higher rates of AA affiliation. Trust in others is an important marker of social capital, which may facilitate access to resources [56] including self-help groups. Furthermore, AA groups encourage self-disclosure and networking with other AA members, both of which women may be more willing to do if they believe that others are more inherently trustworthy.

The theory of interpersonal trust mentions that having high expectancy that others can be relied up on can lead to continious involvement with the group or building relationships [37, 57]. Our finding that women who believed others are trustworthy had higher rates of past year AA affiliation is consistent with what was described in this theory [37].

Our finding that older age was a correlate of past year AA affiliation follows what was previously reported by studies conducted with non-justice-involved population [26, 58]. This in part could be explained in terms of older women having more severe drinking outcomes [58] and experiencing more risks of AUD and by AA affiliation reflecting lifetime AA experiences. Higher number of AUD criteria met also predicted increased past year AA affiliation. This finding is consistent with previous studies that reported experiencing greater consequnces of AUD as a predictor of frequent AA attendance [5, 24]. It is presumed that people seek help for AUD when the consequnces are severe and the problem is disruptive.

In Table 3, AUD self-stigma was positively and significantly associated with limited choice. Women who endorse higher perceived AUD stigma had higher perceived stress and may isolate themselves leading to limited interactions with others including service providers [59]. AUD self-stigma was also negatively associated with self-care and having housing the first night after release. Individuals who perceive stigma may have low self-concept contributing to low self-care. Perceived self-stigma of AUD may also have an adverse effect on housing as women might have limited control and fewer choices regarding housing.

The adjusted model shows a trend toward Non-Hispanic White women, compared to minorities, in reporting higher past-year AA affiliation. Although this association was marginally significant, it might be important to consider racial disparities in future studies [60]. Studies with larger sample size (having enough people per the different race categories) might help to further refine this association. Representation of minority women in the study was appropriate (32.5%) relative to the jail as whole (where 26% of women were from minority groups); however, this is lower than some other jails in the country [61]. Our findings may not be generalizable to other jails in the country because of focus on females only and the lower rates of minority women in the study.

### Strengths and limitations

The study has several strengths. It focused on the correlates of AA affiliation in one of the most disadvantaged groups of the society, justice-involved women with AUD. We targeted women with AUD in pretrial jail detention, the most unstable justice structure often found to be difficult for service planning due to very short (i.e., days) stays [13]. Second, the study examined sets of interrelated individual and interpersonal level variables which have not previously been tested as correlates of AA Affiliation in any population.

This study also has some limitations. The findings reported in this study are based on a cross-sectional data. Longitudinal studies evaluating involvement in 12-step groups would be useful. Second, we enrolled only pretrial jail detainees so our findings may not be generalizable to other segments of the justice system such as women in prison, or to justic-involved men. We also did not control our results for other variables such as other substance usage and previous incarcerations.

## Conclusion

In addition to the known sociodemographic predictors, interpersonal level variables might help holistic understanding of correlates of AA in justice-involved women with AUD. Interventions designed to increase AA attendance among justice-involved women with AUD may benefit from taking into account age, number of AUD criteria met and womens concept of self and others. Introducing AA outreach or alternative interventions for younger, less severely addicted women might improve AUD outcomes. Designing more individualized treatment plan for women who believe others are not trust worthy might help AUD treatment engagement in this population.

## Additional files

Additional file 1: Self-care scale (DOCX 12 kb)

Additional file 2: Limited choice scale (DOCX 13 kb)

## Abbreviations

AA: Alcoholics anonymous
AUD: Alcohol use disorders
DSM-5: Diagnostic and statistical manual-5
GTS: General trust scale
WHO: World Health Organization
