# Supplementary material for: Correlates of alcoholics anonymous affiliation among justice-involved women
Source: BMC Womens Health. 2018 Jul 11;18:125. doi: 10.1186/s12905-018-0614-0 (PMC6042328; doi:10.1186/s12905-018-0614-0)
Supplement: Supplementary file 1 — Self-care scale (DOCX 12 kb) [file 12905_2018_614_MOESM1_ESM.docx]

**Self-Care**

*Please tell me how much you agree or disagree with the following statements:*

| 1. I take good care of myself. | **Scale 24**  (1) Strongly disagree  (2) Disagree  (3) Neither agree or disagree  (4) Agree  (5) Strongly agree | ^1^ |
| --- | --- | --- |
| 2. I need to take care of people around me before I take care of myself. |  | ^2^ |
| 3. I am worth taking care of. |  | ^3^ |
| 4. I am worth protecting. |  | ^4^ |
| 5. I deserve to be able to take care of myself. |  | ^5^ |
| 6. I deserve to protect myself. |  | ^6^ |
| 7. I neglect myself. |  | ^7^ |
| 8. I put myself in dangerous situations |  | ^8^ |
| 9. I take care of my needs. |  | ^9^ |
