# Supplementary material for: Correlates of alcoholics anonymous affiliation among justice-involved women
Source: BMC Womens Health. 2018 Jul 11;18:125. doi: 10.1186/s12905-018-0614-0 (PMC6042328; doi:10.1186/s12905-018-0614-0)
Supplement: Supplementary file 2 — Limited choice scale (DOCX 13 kb) [file 12905_2018_614_MOESM2_ESM.docx]

**Choice**

| **1. How often do you interact with people who drink alcohol or use drugs so that you can get:** | **Scale 16**  (7) daily (7xs/week)  (6) 3 to 6 times a week  (5) once or twice a week  (4) every other week  (3) once a month  (2) less than monthly  (1) one in past 3 months  (0) not in past 3 months |  |
| --- | --- | --- |
| 1a. Housing |  | ^1a^ |
| 1b. Transportation |  | ^1b^ |
| 1c. Money |  | ^1c^ |
|  |  |  |
| **2. How often do you have to interact with people who are risky to your sobriety in order to get:** | **Scale 16**  (7) daily (7xs/week)  (6) 3 to 6 times a week  (5) once or twice a week  (4) every other week  (3) once a month  (2) less than monthly  (1) one in past 3 months  (0) not in past 3 months |  |
| 2a. Housing |  | ^2a^ |
| 2b. Transportation |  | ^2b^ |
| 2c. Money |  | ^2c^ |
| 3. How much choice do you feel like you have in who you associate with, given your need for housing, etc.? | **Scale 17**  (0) None  (1) A little  (2) Some  (3) A great deal | ^3^ |

*People are often told to spend time with people who do not drink or use drugs, and who are supportive and respectful. However, some women feel that they have little choice in who they interact with, given that they need housing, transportation, food, etc. How much does each of the following apply to you:*
